# Reviewed article: Brasil JAI, Raposo LM. Occupational patterns and risks of occupational accidents with biological material: A cross-sectional study, Brazil, 2015-2019. Epidemiol Serv Saude. 2025:34;e20250051

**DOI:** 10.1590/S2237-96222025v34e20250051.a

**Published:** 2025-10-27

**Authors:** Mariana Soares

**Affiliations:** 1 Universidade do Estado de Mato Grosso, Cáceres, MT, Brazil Universidade do Estado de Mato Grosso Cáceres MT Brazil

## Peer review A

## Questionnaire

Does the manuscript contain new and significant information to justify publication? Yes

Does the Abstract (Summary) clearly and accurately describe the content of the article? Yes

Is the problem significant and concisely stated? Yes

Are the methods described comprehensively? Yes

Are the interpretations and conclusions justified by the results? No

Is adequate reference made to other work in the field? Yes

## Manuscript Structure

Length of article is: Adequate

Number of tables is: Adequate

Number of figures is: Adequate

Please state any conflict(s) of interest that you have in relation to the review of this paper. None

Interest: Excellent

Quality: Good

Originality: Average

Overall: Average

Recommendation: Minor Revision

## Comments

De maneira geral, o texto apresenta muitas potencialidades em relação a sua análise estatística e resultados encontrados. Porém algumas informações requerem mudanças em sua forma e escrita do texto.

Em relação ao formato do texto, segundo as normas da ABNT, sugere-se um arquivo que contenha parágrafos justificados.

Resumo: Adequado.

Introdução: Página 1. Linha 56: Trocar o termo acidentes ocupacionais por acidentes de trabalho.

Página 2. Linha 14. “A análise busca oferecer subsídios que contribuam para o desenvolvimento e a implementação de estratégias preventivas mais eficazes, promovendo ambientes de trabalho mais seguros e saudáveis para os trabalhadores”. Sugiro levar o parágrafo para antes do objetivo, como uma forma de justificar a importância do trabalho.

Metodologia: Página 2. Linha 33. Os autores evidenciam na metodologia que a análise permitiu identificar a distribuição geográfica e temporal, porém não foi realizado nenhum mapa ou ilustrações gráficas que permitisse inferir essa informação “geográfica”, apenas uma categorização por regiões brasileiras, também não foi realizada uma análise de tendência temporal anual, somente o conglomerado entre os anos de 2015 a 2019, por esses motivos, sugiro retirar essa informação.

Página 2. Linha 41. Incluir a flexão de gênero, da população do estudo, trabalhadores(as), conforme a Política Nacional de Saúde do trabalhador(a). Repetir a flexão em todas as vezes que foi mencionado no manuscrito.

Resultados: Página 4 e 5. Os parágrafos que descrevem são a Figura 1 e Tabela 2 tornam-se repetitivos e de difícil leitura, com informações truncadas. Sugiro uma nova redação do texto, descrevendo a Figura 1, concomitante com a Tabela 2.

Na Tabela 1. Como se trata de uma categorização descritiva das informações, recomendo que os autores(as) coloquem todas as informações de: sexo (feminino/masculino, raça/cor (5 categorias) e categoria de trabalho (formal e informal). Conforme descreveram na metodologia.

Além disso, os autores(as) não explicam no texto o porquê da distinção da raça branca/não branca. Existe alguma explicação plausível para comparação entre os grupos?

Na figura 1. Mudar tamanho e fonte da figura. Título extenso, sugiro retirar a classificação da categoria do título e explicar/descrever nos resultados encontrados, ou mesma na metodologia.

Discussão: Página 6. Linha 3. Sugiro levar o parágrafo com as limitações identificadas no texto para o final do texto.

Página 7. Linha 38 “No Grupo 1, o uso parcial dos dispositivos deve-se à ausência de treinamentos ou equipamentos adequados”, acredito que não seja possível inferir essa informação, visto que foi realizado no texto uma análise de dados secundários, ao qual não foram questionados sobre essa informação.

